# Assessment of nutritional status of critically Ill pediatric patients and its impact on outcome in a low-and middle-income settings

**DOI:** 10.1007/s00431-026-07420-5

**Published:** 2026-10-07

**Authors:** Aya Osama Mohamed, Asmaa Farrag El Sayed, Dina Mohamed Hassan, Shereen A. Mohamed

**Affiliations:** 1 Pediatrics, Faculty of Medicine, Kasralainy, Cairo University, Cairo, Egypt; 2 Pediatric Intensive Care Unit, Cairo University Children Hospital, Cairo, Egypt; 3 Pediatric Intensive Care Unit, Cairo University Mounira Pediatric Hospital (Abou El Riche), Sayeda Zeinab, Kasr Al Aini, PO Box: 11562, Cairo, Egypt; 4 Public Health and Community Medicine Department, Faculty of Medicine, Kasralainy, Cairo University, Cairo, Egypt

**Keywords:** Malnutrition, PYMS, STRONGkids, PRISM, PICU

## Abstract

Malnutrition among children is a crucial public health issue. Children in critical care units may suffer from nutritional vulnerability due to their underlying disease or barriers to nutrient delivery. Early detection and management of malnutrition may improve clinical outcomes. To evaluate the nutritional status of critically ill children admitted to the pediatric intensive care unit (PICU) and its prognostic outcome. This observational prospective cohort research entailed 250 children admitted to the PICU. All participants were assessed at admission and weekly thereafter for 28 days. Nutritional status was evaluated using anthropometric measurements and the PYMS and STRONGkids scores. The PRISM score was used to assess illness severity on the day of admission. 59.2% of patients were underweight and/or stunted at admission and 59% at day 28 of follow-up. PYMS, and STRONGkids scores were significantly higher among underweight and/or stunted on day 1 and during follow-up (*p* < 0.05). Multivariable regression analysis demonstrated that admission PRISM score was an independent predictor of mortality (aOR 1.50, 95% CI 1.19–1.88, *p* = 0.001), mechanical ventilation requirement (aOR 1.18, 95% CI 1.07–1.31, *p* = 0.001), and feeding interruptions (aOR 1.29, 95% CI 1.16–1.45, *p* < 0.001). Socioeconomic status was independently associated with feeding interruptions and length of stay.

*Conclusion*: Underweight and/or stunting was common among PICU patients. PYMS and STRONGkids were effective in identifying nutritional risk. After adjustment for confounding variables, clinical severity rather than baseline malnutrition independently predicted adverse clinical outcomes.

**Table:** 

**What is Known:** • *Underweight and/or stunting were prevalent among children admitted to the PICU and this represent a crucial public health problem.* • *Both PYMS and STRONGkids were useful screening tools for identifying children at risk of malnutrition.*	
**What is New:** • *This study demonstrated that clinical severity and the need for inotropic support were the primary independent predictors of adverse outcomes.* • *The baseline malnutrition was not independently associated with mortality, mechanical ventilation, feeding interruptions, or length of PICU stay.*

**Supplementary Information:**

The online version contains supplementary material available at https://doi.org/10.1007/s00431-026-07420-5.

## Introduction

Nutritional status is considered one of the cardinal factors influencing the outcomes of life-threatening diseases in children. Malnutrition incidence among children in the pediatric intensive care unit (PICU) is reported as high as 35–72% [1].

Nutritional support is a fundamental component of children’s health that resists the deleterious sequelae of critical illness by providing energy and nutritional substrates essential for protein synthesis, limiting the loss of lean body mass [2], and regulating the body immune function [3]. Consequently, if malnutrition is overlooked, the prognosis of these patients may be dramatically affected.

The American Society for Parenteral and Enteral Nutrition (ASPEN) recommends using rapid and easy nutritional assessment methods to detect risk of malnutrition, such as the Screening Tool for Risk of Nutritional Status and Growth (STRONG Kids) and the Pediatric Yorkhill Malnutrition Score (PYMS) [4].

Malnutrition is highly prevalent in developing nations and remains a major health hazard. Challenges persist in these countries despite the notable global progress made in handling the causes of malnutrition. Limited evidence exists regarding the multifactorial determinants of malnutrition in critically ill children, including the socio-economic, cultural, and ecological factors [5]. Therefore, this study was conducted to assess the nutritional status of children admitted to the pediatric intensive care unit (PICU), to examine the relationship between growth parameters and clinical outcomes, and to determine the proportion of non-survivors among malnourished patients.

### Patients and methods

This prospective observational cohort study was carried out in the pediatric intensive care units (PICUs) of Cairo University Children’s Hospital from November 2023 to March 2025.

Inclusion criteria included all patients aged 1 month to 14 years enrolled in the PICU throughout the duration of the study. Exclusion criteria were patients with chromosomal anomalies, infants who were small for gestational age (birth weight < 2.5 kg), and patients who died within 24 h of admission.

On admission, socioeconomic status of the patients was performed using the formerly validated socioeconomic status scale [6]. It included seven elements with an entire score of 84 points: education, occupation, family (residence and number), family possession, and home sanitation (involving crowding index), and economic and health care domains (usual source of care). The score was then classified into quartiles (< 21, 21 to < 42, 42 to < 63, and 63–84), corresponding to very low, low, middle, and high socioeconomic status, respectively. The Pediatric Risk of Mortality (PRISM) score was used to assess illness severity in the PICU [7]. It was calculated for all patients within 24 h of admission.

The Screening Tool for Risk of Nutritional status and Growth (STRONGkids) was used to evaluate the malnutrition risk in children; it includes a subjective global assessment, illness vulnerability, nutritional intake, and body weight waste/gain ratio. Children with a score of 0 were classified as being at low risk of malnutrition, those with a score of 1–3 at moderate risk, and those with a score of 4–5 at high risk (maximum score, 5 points) [3]. The Pediatric Yorkhill Malnutrition Score (PYMS) was also calculated; this pediatric screening tool consists of four questions, with a maximum score of 7 points, assessing the child’s anthropometric measurements and nutritional balance. A score of 0 indicated low risk, a score of 1 medium risk, and a score of ≥ 2 high risk [3].

Patients were classified as underweight if the weight-for-age Z score was <  − 2, stunted if the height-for-age Z score was <  − 2, and wasted if the weight-for-height Z score was <  − 2 [8].

Hospital-acquired malnutrition was diagnosed when the weight-for-age Z score was normal at admission (normal weight) and then became below − 2 (underweight) during follow-up [9, 10].

STRONGkids, PYMS, anthropometric measures, and laboratory investigations, including complete blood count (CBC), blood urea nitrogen (BUN), creatinine (Cr), aspartate aminotransferase (AST), alanine aminotransferase (ALT), C-reactive protein (CRP), and serum albumin, were documented on days 1, 7, 14, 21, and 28 of admission.

The study protocol was approved by the Research Ethics Committee (REC) of the Faculty of Medicine, Cairo University (approval code: MS-542–2023).

### Statistical analysis

Categorical variables were expressed as frequencies and percentages and analyzed using the chi-square test.

Quantitative variables were expressed as mean ± standard deviation. The Shapiro–Wilk test was used to test the normality. Student’s *t*-test was used to analyze normally distributed variables. Non-normally distributed data were expressed as medians and interquartile ranges (IQRs) and analyzed using the non-parametric Mann–Whitney *U* test. A value of *p* < 0.05 was considered statistically significant.

Multivariable binary logistic regression analyses were performed to identify independent predictors of mortality, mechanical ventilation, and feeding interruptions. Variables entered in the models were selected based on clinical relevance and univariate analysis results. Adjusted odds ratios (aORs) with 95% confidence intervals (CIs) were reported. Model fit was assessed using the Hosmer–Lemeshow goodness-of-fit test, and explained variance was evaluated using Nagelkerke *R*^2^.

A multivariable linear regression model was conducted to assess independent predictors of length of PICU stay. Because length of stay was not normally distributed, it was log-transformed prior to analysis to satisfy model assumptions. Unstandardized beta coefficients (*β*) with 95% confidence intervals were reported.

Multicollinearity was assessed prior to model construction. A *p*-value < 0.05 was considered statistically significant. Statistical analyses were performed using SPSS version 22.0.

## Results

A total of 250 patients were enrolled in the study; 138 (55.2%) were males and 112 (44.8%) were females, with a median age of 19 months.

Regarding parents’ education, 131 (52.4%) were university graduates or postgraduates. One hundred sixty-six cases (66.4%) were of the middle socioeconomic class, and 68 cases (27.2%) were of very low or low socioeconomic class according to El-Gilany’s socioeconomic scoring system. Table 1 shows the causes of admission of the studied patients; pneumonia was the leading cause (69 cases, 27.6%), and 37/69 (53.6%) of these patients were underweight and/or stunted.

**Table 1:** Diagnoses of the studied patients admitted to the PICU

Diagnosis	Count (%)
Pneumonia	69 (27.6%)
Septic shock	30 (12.0%)
Post operative	22 (8.8%)
DCL*	21 (8.4%)
Guillain–Barré syndrome	15 (6.0%)
CHD**	12 (4.8%)
Pneumonia and CHD	11 (4.4%)
Foreign body aspiration	10 (4.0%)
Status epilepticus	9 (3.6%)
Hypovolemic shock	1 (0.4%)
Others	50 (20.0%)
**Total**	**250** (**100%**)

^*^DCL, disturbed conscious level; **CHD, congenital heart disease

**Fig. 1:**
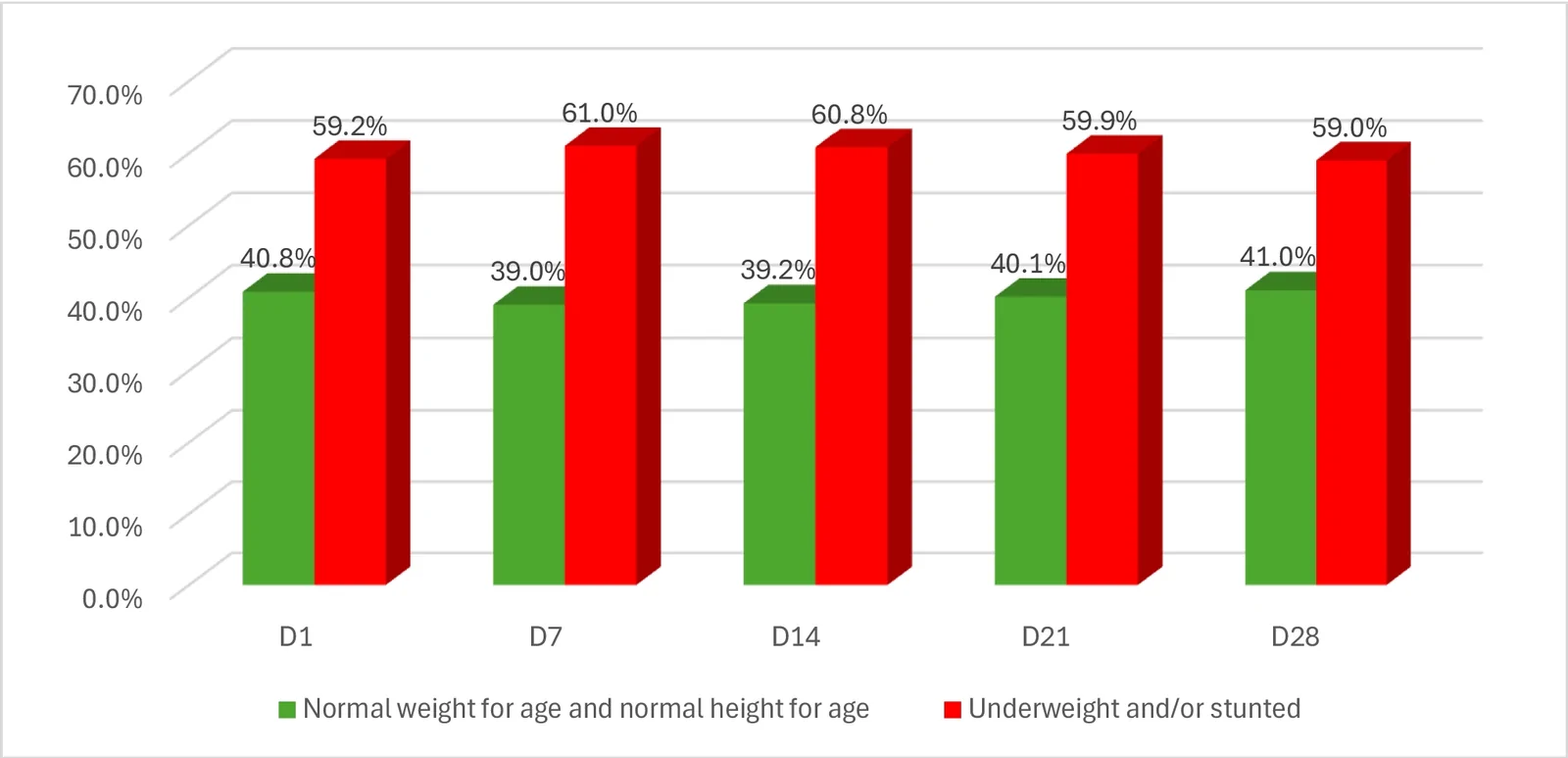
Percentage of normal weight for age and normal height for age; versus underweight and/or stunted patients on day 1, 7, 14, 21, and 28 of admission during PICU stay

Table 2 illustrates the anthropometric assessment of the studied patients on days 1, 7, 14, 21, and 28 of admission, and Table 3 shows the nutrition screening tools and PRISM score of the studied patients across days 1, 7, 14, 21, and 28. On admission, 148/250 (59.2%) patients were underweight and/or stunted; 152/249 (61.0%) on day 7; 146/240 (60.8%) on day 14; 142/237 (59.9%) on day 21, and 135/229 (59.0%) on day 28, with no significant change during follow-up, as shown in Fig. 1.

**Table 2:** Anthropometric assessment of the studied patients on days 1, 7, 14, 21, and 28 of admission to the PICU

	Day 1 (*n* = 250)	Day 7 (*n* = 249)	Day 14 (*n* = 240)	Day 21 (*n* = 237)	Day 28 (*n* = 229)
Weight For Age z score Median, IQR	− 1.15 (− 2.6: − 0.1)	− 1.2 (-1.2:-0.1225)	− 1.2 (-2.8:-0.25)	− 1.2 (-2.8:-0.18)	− 1.1 (-2.7:-0.088)
Height For Age z score Median, IQR	− 2.1 (-3.2:-0.8)	− 2.1 (-3.3:-0.8)	− 2.2 (-3.325:-.09)	−2.1 (-3.3:-0.8)	− 2.2 (-3.2:-0.9)
Weight For Height z score Median, IQR	0.45 (-1.5:1.8)	0.5 (-1.5:1.625*)*	0.42 (-1.525:-1.6)	0.4 (-1.7:1.6)	0.5 (-1.3:1.9)

Interquartile range (IQR)

**Table 3:** Nutrition screening tools and PRISM score of the studied patients on days 1, 7, 14, 21, and 28 of admission to the PICU

	Day 1 (*n* = 250)	Day 7 (*n* = 249)	Day 14 (*n* = 240)	Day 21 (*n* = 237)	Day 28 (*n* = 229)
STRONG kids score Median, IQR	2 (1–3)	2 (1-3)	2 (0-3)	1 (0-2.25)	0 (0-2)
PYMS score Median, IQR	2 (2–4)	2 (1–4)	2 (0-3)	1 (0-3)	0 (0-2)
PRISM score Median, IQR	2 (5)

The admission PRISM score of underweight and/or stunted patients ranged between 0.00 and 14.00 (median, 3.00), while that of patients with normal weight for age and normal height for age ranged between 0.00 and 12.00 (median, 1) (*p* = 0.002). The fatality rate among the studied patients was 8.4%.

Regarding routine laboratory tests (CBC, BUN, Cr, AST, ALT, CRP, and serum albumin) on days 1, 7, 14, 21, and 28, comparing patients with normal weight for age and normal height for age versus underweight and/or stunted patients, only ALT differed significantly, on days 1, 7, 21, and 2.

Eighty-nine of the 250 patients (35.6%) had feeding interruptions. Five of these 89 patients (5.6%) developed hospital-acquired malnutrition (weight-for-age Z score was normal at admission then became below − 2 during follow-up) during the PICU stay (*p* = 0.101).

Regarding medical interventions, 95/250 (38%) patients required mechanical ventilation, of whom 68 (71%) were underweight and/or stunted which was statistically different from the normal weight for age and normal height for age (*p* = 0.018); however, there was no significant difference in the duration of mechanical ventilation between the two groups (*p* = 0.664).

While 65/250 (26%) needed inotropic support, 47/65 (72.3%) were underweight and/or stunted, with statistically different from normal weight for age and normal height for age patients (*p* = 0.047) (Table 4). There was non-significant relation between state of nutrition and PICU stay duration (*p* = 0.447).

**Table 4:** Medical interventions of the studied patients during the PICU stay

	Yes *n* (%)	No *n* (%)	Total *n* (%)
Mechanical ventilation	95 (38%)	155 (62%)	250 (100%)
Inotropic support	65 (26%)	185 (74%)	250 (100%)
Duration of mechanical ventilation (days) Median, IQR	6 (3–14)		
PICU length of stay (days) Median, IQR	13 (7–21)		

Sixty-five of the 250 patients (26%) required inotropic support, of whom 47 (72.3%) were underweight and/or stunted, with a statistical difference from normal weight for age and normal height for age (*p* = 0.047) (Table 4). There was no significant relation between nutritional status and the duration of PICU stay (*p* = 0.447).

STRONGkids and PYMS scores of patients with normal weight for age and normal height for age versus underweight and/or stunted patients on day 1 of admission and during the PICU stay are shown in Table 5.

**Table 5:** STRONG kids and PYMS scores versus nutritional status of the studied patients on day 1 of admission during PICU stay

	Normal weight for age and normal height for age (*n* = 102)		Underweight and/or stunted (*n* = 148)		***p*** value
	Median	IQR	Median	IQR	
STRONG kids score (D1)	1.00	0–3	3.00	1–4	< 0.001
PYMS score (D1)	2.00	0.25–2	4.00	2–5	< 0.001

Additionally, Table 6 shows the relationship between the nutritional status and socioeconomic status of the studied patients.

**Table 6:** Relationship between the nutritional status and socioeconomic status of the studied patients

Socioeconomic status	Nourished count (%)	Malnourished count (%)	*p* value
High	13 (12.75%)	3 (2.03%)	< 0.001
Middle	83 (81.37%)	83 (56.08%)	
Low	6 (5.88%)	42 (28.38%)	
Very low	0 (0%)	20 (13.51%)	
Total	**102 (100%)**	**148 (100%)**	

Multivariable regression analysis demonstrated that admission PRISM score was an independent predictor of mortality (aOR 1.50, 95% CI 1.19–1.88, *p* = 0.001), mechanical ventilation (aOR 1.18, 95% CI 1.07–1.31, *p* = 0.001), and feeding interruptions (aOR 1.29, 95% CI 1.16–1.45, *p* < 0.001). Requirement for inotropic support significantly increased the odds of mortality (aOR 20.00, 95% CI 2.33–166.67, *p* = 0.006) and mechanical ventilation (aOR 2.86, 95% CI 1.35–6.06, *p* = 0.006). Socioeconomic status was independently associated with feeding interruptions and length of stay. After adjustment for clinical severity and demographic factors, baseline malnutrition was not independently associated with mortality, mechanical ventilation, feeding interruptions, or length of PICU stay (for full details of the regression models are provided in the supplementary tables).

## Discussion

Malnutrition is a major widespread health issue in children, particularly in developing nations. Nearly 50% of non-survivors below five years old were suffering from inadequate nourishment [11].

This cohort analytical study included 250 patients admitted to the PICU for evaluating their state of nutrition and its prognostic outcome.

Pneumonia was the major cause of admission 27.6% of the studied patients, in accordance with other studies [12–15], underscoring that pneumonia is a serious threat to children’s health.

The current study revealed that 53.6% of pneumonia patients were underweight and/or stunted, and that was highlighted in other studies [16, 17], even reporting that it increases the risk of death [18, 19].

Clinical assessment of the children was done every week for 28 days using the z score growth chart.

Evaluation of nutritional status by these anthropometric measurements revealed that 59.2% of patients were underweight and/or stunted on admission and at day 28 of follow-up, with no significant difference in percentage during follow-up, and this high percentage may be explained by the fact that this study was conducted in a low- and middle-income setting. Moreover, patients seeking care at a specialized tertiary center often suffer from severe illness. Moreover, patients seeking care at a specialized tertiary center often have more severe illness. In accordance with these results, a recent study from India by Jain et al. reported a malnutrition incidence of 51.9% among severely ill children [20]. In addition, a study by Campos-Miño et al. in Latin American countries reported that underweight and length-for-age below − 2 Z scores were present in 47.7% of children in the PICU [21].

In contrast to our results, a study by Wong Vega et al. in the USA found that malnutrition prevalence was 19.7% upon admission [22]. This variation may be clarified by the higher prevalence of malnutrition in low- and middle-income settings.

In our study, STRONG kids was used as an assessment method for detecting malnutrition risk, and its scores were significantly higher in underweight and/or stunted patients than in those with normal weight for age and normal height for age on day 1 and during follow-up (*p* < 0.05). In agreement with these results, Wang et al. concluded that STRONGkids is a simple tool for detecting the risk of malnutrition in sick children [23].

In the current study, the PYMS score was used as another screening tool and revealed a significant difference between underweight and/or stunted children and those with normal weight for age and normal height for age children at day 1 and during follow-up (*p* < 0.05). In agreement with these results, a study conducted by Malekiantaghi et al. found a significant correlation between the PYMS and Z score growth curves for weight -for-height and BMI-for-age Z-scores [24]. Seremet Kurklu et al. compared these nutrition screening tools (STRONGkids, PYMS, and PNST) and reported that the PYMS score is useful for detecting malnutrition risk in hospitalized children, as it exhibited favorably high number of sensitivities [90.9% and 84.6%] for weight for age and BMI, respectively [3]. The PYMS is a validated questionnaire used as a method for malnourishment assessment in different world regions [24].

In the present study, although underweight and/or stunting and higher nutritional risk scores were associated with mechanical ventilation need in univariate analysis, multivariable regression demonstrated that after adjustment for confounding factors, baseline malnutrition was not independently associated with the need for mechanical ventilation.This was in accordance with Toh et al. who studied the variation in mechanical ventilation period between normal-weight, underweight, overweight, and obese patients in the PICU, and found no significant difference [25]. This contrasts with Bechard et al. who found that underweight cases needed a longer duration of mechanical ventilation than normal-weight patients [26].

In the current study, 26% of the admitted patients required inotropic support, and there was a significant correlation among underweight and/or stunted upon admission and the need for inotropic support (*p* = 0.047). Samanta et al. also reported that there was a significant correlation between weight for age on admission and inotropic support in children admitted to the PICU. Moreover, children with a BMI between the 25th and 50th percentiles in their study had lower inotrope requirements [27].

There was a strong relation among the state of nutrition and socioeconomic status (*p* < 0.001), as our study showed that a high proportion of malnourished patients (41.89%) were of low (28.38%) and very low (13.51%) socioeconomic status.

Consistent with our results, a study by Ahmad et al. in Pakistan reported that children in the lowest affluence category had increased rate of malnutrition (stunting, wasting, and underweight) than those in higher affluence category, which was primary related to lowered affordability of healthcare, quality nutrition, and hygiene [28]. Tadesse et al. in Ethiopia also reported similar findings [29]. Most developing countries suffer from malnutrition due to socioeconomic issues [30].

A multivariable binary logistic regression was performed to evaluate the predictors of feeding interruptions, and it was observed that low socioeconomic status was independently associated with significantly lower odds of feeding interruptions compared to the moderate group (aOR, 0.46, *p* = 0.034. A linear model was performed to evaluate the independent association between baseline characteristics, clinical severity, and length of PICU stay, socioeconomic status was a significant predictor (*p* = 0.009), with patients in the low socioeconomic category demonstrating significantly shorter lengths of stay compared to the moderate group (*p* = 0.002).

Hematological abnormalities as a sequela of malnutrition including anemia and thrombocytopenia, were statistically significant in the current study (*p* 0.010 and *p* < 0.00, respectively). This observation agrees with several other studies [31–37].

This may be attributed to the malnutrition effect on the hematopoietic system, leading to defective production of all blood cell lines [37].

The PRISM score is accounted as one of the best predictors of mortality risk [38], and in this study, we found the mean PRISM score was markedly greater in non-survivors than in those who survived (*p* < 0.001). This was confirmed by the multivariable binary logistic regression conducted to determine the independent predictors of PICU mortality which revealed that PRISM score and the need for inotropic support were the only independent predictors of mortality.

Other studies reported similar results, finding that PRISM scores were high among non-survivors [7, 12, 39].

The mortality rate in the present study was 8.4%, and nearly equivalent rates were reported in other studies [40, 41].

Furthermore, in addition to clinical data, ICU expertise, and resources, the nutritional state is considered a factor associated w22ith mortality [42]. In our study, non-survivors had a higher mean PRISM score than survivors. This is supported by the significant association between mortality and higher STRONGkids and PYMS scores (*p* < 0.05). However, there is no significant difference in mortality between underweight and/or stunted patients and those with normal weight for age and normal height for age (10.3% vs. 5.3%, respectively; *p* > 0.05).

Other studies have examined the correlation between mortality and nutritional status: Nangalu et al. reported that mortality was significantly higher in severely malnourished subjects [43]. Morales et al., in agreement with our results, reported that all non-survivors presented with high PYMS scores [44]. Similarly, Desai et al. found that children with prolonged malnutrition and wasting had a higher mortality rate [45].

This relationship between malnutrition and poor outcomes may be explained by a diminished metabolic reserve accompanied by a highly catabolic state, inadequate nutritional support, or malabsorption [46]. Hence, in underweight children, nutritional rehabilitation takes equal, if not greater, priority than controlling the disease process; in children with a healthy weight, however, nutritional rehabilitation is not usually the primary concern of the pediatrician. Nevertheless, irrespective of the initial nutritional status of the patient, enteral nutrition should be initiated promptly, as recommended by the 2021 Surviving Sepsis Campaign guidelines [27]. Thus, nutritional support should be an integral part of a multidisciplinary approach to managing critically ill pediatric patients.

This study has certain limitations, including its relatively small sample size and single-center design, which may limit the generalizability of the findings. Larger, multicenter longitudinal studies are needed to confirm these results. Given the observed prevalence of undernutrition and its association with illness severity in unadjusted analyses, nutritional assessment at PICU admission may be beneficial. Further longitudinal research incorporating larger cohorts, diverse assessment tools, and longer follow-up durations is required to better define the independent clinical impact of malnutrition in critically ill children.

## Conclusion

Underweight and/or stunting were prevalent among children admitted to the PICU. Both PYMS and STRONGkids were useful screening tools for identifying children at risk of malnutrition. Although underweight and/or stunting and higher nutritional risk scores were associated with mortality in univariate analysis, multivariable regression demonstrated that clinical severity (PRISM score) and the need for inotropic support were the primary independent predictors of adverse outcomes. After adjustment for confounding factors, baseline malnutrition was not independently associated with mortality, mechanical ventilation, feeding interruptions, or length of PICU stay.

## Supplementary Information

Below is the link to the electronic supplementary material.

ESM 1 (DOCX 23.9 KB)

## Data Availability

The data sets generated and analyzed during the current study are available from the corresponding author.
