# Supplementary material for: Assessment of nutritional status of critically Ill pediatric patients and its impact on outcome in a low-and middle-income settings
Source: Eur J Pediatr. 2026 Oct 7;185(11):810. doi: 10.1007/s00431-026-07420-5 (PMC13645988; doi:10.1007/s00431-026-07420-5)
Supplement: Supplementary file 1 [file 431_2026_7420_MOESM1_ESM.docx]

**Supplementary tables for regression analysis**

**Mechanical ventilation:**

A multivariable binary logistic regression was performed to ascertain the independent predictors of patients requiring mechanical ventilation. The model was statistically significant, chi squared = 48.649, p < .001, demonstrating good fit (Hosmer-Lemeshow p = .335) and explaining 24.1% (Nagelkerke R squared) of the variance.

Higher admission PRISM scores (aOR = 1.18; 95% CI: 1.07–1.31, p = .001) and the requirement for inotropic support (aOR = 2.86; 95% CI: 1.35–6.06, p = .006) independently and significantly increased the odds of needing mechanical ventilation. Baseline nutrition status, age, and socioeconomic status did not contribute to the model's predictive ability.

**Supplementary Table 1. Multivariable Logistic Regression of Predictors for Mechanical Ventilation**

| **Variable** | **β** | **SE** | **Adjusted Odds Ratio (aOR)** | **95% CI for aOR** | **p-value** |
| --- | --- | --- | --- | --- | --- |
| **Age (months)** | 0.004 | 0.003 | 1.00 | 1.00 – 1.01 | 0.218 |
| **PRISM Score** | 0.169 | 0.053 | 1.18 | 1.07 – 1.31 | 0.001* |
| **Inotropic Support (Yes vs. No)** | 1.049 | 0.383 | 2.86 | 1.35 – 6.06 | 0.006* |
| **Nutrition (Well Nourished vs. Underweight/Stunted)** | -0.509 | 0.329 | 0.60 | 0.32 – 1.15 | 0.122 |
| **Socioeconomic Status** |  |  |  |  | 0.687 |
| **quad Very Low vs. Moderate (Ref)** | 0.286 | 0.655 | 1.33 | 0.37 – 4.81 | 0.662 |
| **quad Low vs. Moderate (Ref)** | -0.170 | 0.347 | 0.84 | 0.43 – 1.67 | 0.625 |

**Mortality:**

A multivariable binary logistic regression determined the independent predictors of PICU mortality. The overall model was statistically significant, chi squared = 65.792, p < .001, with excellent goodness-of-fit (Hosmer-Lemeshow p = .976), explaining 52.8% (Nagelkerke R squared) of the variance.

The admission PRISM score and the need for inotropic support were the only independent predictors of mortality. For every 1-point increase in PRISM score, the odds of mortality increased by 1.50 times (p = .001). Patients requiring inotropes had 20.00 times higher odds of mortality compared to those who did not (p = .006). Very Low socioeconomic status approached, but did not reach, statistical significance (p = .058).

**Supplementary Table 2. Multivariable Logistic Regression of Predictors for Mortality**

| **Variable** | **β** | **SE** | **Adjusted Odds Ratio (aOR)** | **95% CI for aOR** | **p-value** |
| --- | --- | --- | --- | --- | --- |
| **Age (months)** | -0.008 | 0.008 | 0.99 | 0.98 – 1.01 | 0.340 |
| **PRISM Score** | 0.404 | 0.117 | 1.50 | 1.19 – 1.88 | 0.001* |
| **Inotropic Support (Yes vs. No)** | 3.004 | 1.101 | 20.00 | 2.33 – 166.67 | 0.006* |
| **Nutrition (Well Nourished vs. Underweight/Stunted)** | -0.240 | 0.716 | 0.79 | 0.19 – 3.21 | 0.738 |
| **Socioeconomic Status** |  |  |  |  | 0.058 |
| **quad Very Low vs. Moderate (Ref)** | 2.756 | 1.454 | 15.63 | 0.91 – 250.00 | 0.058 |
| **quad Low vs. Moderate (Ref)** | -0.516 | 0.690 | 0.60 | 0.15 – 2.31 | 0.455 |

**Feeding Interruption:**

A multivariable binary logistic regression evaluated predictors of feeding interruptions. The model was statistically significant, chi squared = 71.154, p < .001, demonstrating excellent goodness-of-fit (Hosmer-Lemeshow p = .755) and explaining 34.0% (Nagelkerke R squared) of the variance.

Higher admission PRISM scores (p < .001) and the requirement for inotropic support (p = .008) independently increased the odds of experiencing feeding interruptions. Subgroup analysis of socioeconomic status revealed that patients in the Low socioeconomic category had significantly lower odds of feeding interruptions compared to the Moderate reference group (aOR = 0.46; 95% CI: 0.22–0.94, p = .034). Baseline malnutrition was not an independent predictor.

**Supplementary Table 3. Multivariable Logistic Regression of Predictors for Feeding Interruption**

| **Variable** | **β** | **SE** | **Adjusted Odds Ratio (aOR)** | **95% CI for aOR** | **p-value** |
| --- | --- | --- | --- | --- | --- |
| **Age (months)** | 0.004 | 0.004 | 1.00 | 1.00 – 1.01 | 0.239 |
| **PRISM Score** | 0.257 | 0.057 | 1.29 | 1.16 – 1.45 | < .001* |
| **Inotropic Support (Yes vs. No)** | 1.052 | 0.396 | 2.87 | 1.32 – 6.21 | 0.008* |
| **Nutrition (Well Nourished vs. Underweight/Stunted)** | 0.084 | 0.353 | 1.09 | 0.54 – 2.17 | 0.813 |
| **Socioeconomic Status** |  |  |  |  | 0.104 |
| **quad Very Low vs. Moderate (Ref)** | -0.538 | 0.699 | 0.58 | 0.15 – 2.30 | 0.442 |
| **quad Low vs. Moderate (Ref)** | -0.785 | 0.370 | 0.46 | 0.22 – 0.94 | 0.034* |

**Length of PICU stay:**

A Linear Model was performed to evaluate the independent association between baseline characteristics, clinical severity, and length of PICU stay. To satisfy the assumption of normality for linear regression, the length of stay variable was log-transformed prior to analysis. The overall multivariable model was statistically significant (F(6, 243) = 8.512, p < .001) and accounted for 17.4% of the variance in the log-transformed length of stay.

Baseline malnutrition was not significantly associated with length of stay (p = .403). The admission PRISM score was a strong independent predictor (p = .001); for every 1-point increase in the PRISM score, the expected length of stay increased by approximately 5.7%. Age was also independently associated with a slight increase in length of stay (p = .010). Furthermore, socioeconomic status was a significant predictor (p = .009), with patients in the Low socioeconomic category demonstrating significantly shorter lengths of stay compared to the Moderate reference group (p = .002). The need for inotropic support approached, but did not reach, statistical significance (p = .058).

S**upplementary Table 4. Multivariable General Linear Model of Predictors for Length of Stay**

| **Variable** | **Unstandardized β** | **SE** | **95% CI for β** | **p-value** |
| --- | --- | --- | --- | --- |
| **Age (months)** | 0.003 | 0.001 | 0.001 – 0.005 | 0.010* |
| **PRISM Score** | 0.056 | 0.016 | 0.024 – 0.089 | 0.001* |
| **Inotropic Support** |  |  |  |  |
| **quad Yes vs. No (Ref)** | 0.238 | 0.125 | -0.008 – 0.485 | 0.058 |
| **Nutrition Status** |  |  |  |  |
| **quad Well Nourished vs. Underweight/Stunted (Ref)** | 0.082 | 0.098 | -0.110 – 0.274 | 0.403 |
| **Socioeconomic Status** |  |  |  | 0.009* |
| **quad Very Low vs. Moderate (Ref)** | -0.314 | 0.202 | -0.712 – 0.085 | 0.123 |
| **quad Low vs. Moderate (Ref)** | -0.330 | 0.107 | -0.540 – -0.119 | 0.002* |

*CI = Confidence Interval; SE = Standard Error. The dependent variable is log-transformed Length of Stay.*

** Indicates statistical significance (p < .05).*
